# Basic Physicochemical Properties of Pectin from Underutilized Chili Peppers Subjected to Different Heating Regimes

**DOI:** 10.3390/foods15010118

**Published:** 2025-12-31

**Authors:** Olivia Patricia Ramos-Aguilar, Saul Ruiz-Cruz, Juan Ornelas-Paz, José de Jesús Ornelas-Paz, Jaime David Pérez-Martínez, Claudio Rios-Velasco, Carmen Lizette Del-Toro-Sánchez, Enrique Márquez-Ríos, Irma Ofelia Maya-Meraz, Vrani Ibarra-Junquera, Pilar Escalante-Minakata, José Juan Virgen-Ortiz

**Affiliations:** 1 Coordinación de Fisiología y Tecnología de Alimentos de la Zona Templada, Centro de Investigación en Alimentación y Desarrollo A.C., Av. Río Conchos S/N, Parque Industrial, Ciudad Cuauhtémoc 31570, Chihuahua, Mexico; opramosa@hotmail.com (O.P.R.-A.); juanopmx@gmail.com (J.O.-P.); claudio.rios@ciad.mx (C.R.-V.); 2 Departamento de Investigación y Postgrado, Universidad de Sonora, Blvd. Rosales y Luis Encinas S/N, Hermosillo 83000, Sonora, Mexico; saul.ruizcruz@unison.mx (S.R.-C.); carmen.deltoro@unison.mx (C.L.D.-T.-S.); enrique.marquez@unison.mx (E.M.-R.); 3 Facultad de Ciencias Químicas, Universidad Autónoma de San Luis Potosí, Manuel Nava 6 Zona Universitaria, San Luis Potosí 78210, San Luis Potosí, Mexico; jdavidperez@uaslp.mx; 4 Facultad de Ciencias Agrotecnológicas, Universidad Autónoma de Chihuahua, Av. Universidad S/N, Ciudad Universitaria, Chihuahua 31110, Chihuahua, Mexico; imaya@uach.mx; 5 Facultad de Ciencias Químicas, Universidadde Colima, Km. 9 Carretera Coquimatlán-Colima, Coquimatlán 28400, Colima, Mexico; vij@ucol.mx (V.I.-J.); minakata@ucol.mx (P.E.-M.); 6 Centro de Investigación en Alimentación y Desarrollo, A.C., Centro de Innovación y Desarrollo Agroalimentario de Michoacán, A.C., Km. 8 Antigua Carretera a Pátzcuaro S/N, Morelia 58341, Michoacán, Mexico; jose.virgen@ciad.mx

**Keywords:** new pectin sources, chili, functional polysaccharides, structural analysis

## Abstract

Brown Jalapeno peppers (BPs) are accidentally harvested and discarded at different points of the postharvest handling and processing lines because they are not visually attractive to consumers. Water-, chelator-, and alkali-soluble pectins (WSP, CSP, and NSP) were extracted from raw and heat-treated BPs and evaluated for basic physicochemical properties. Heat treatment increased the yield of WSP with the concomitant decrease in CSP and NSP. The extracted pectins were classified as low-methoxyl pectins, as only CSP from raw peppers showed a degree of methyl esterification slightly above 50%. Heat treatment decreased (13.5–86.2%) the molecular weight of the most abundant fraction in all pectins. It also decreased the degree of methyl esterification and Gal and Man contents in all pectins. The viscosity of WSP solutions decreased (28–41%) with heat treatment. Heat treatment negatively affected the color of pectin.

## 1. Introduction

Mexico is the second producer and the largest exporter of fresh peppers in the world, with Jalapeno peppers being the most produced [1,2]. Jalapeno peppers are intended to be harvested exclusively when ripe and have a homogeneous green or red external color [3]. However, many peppers showing a homogeneous brown color are harvested along with green or red pods. The brown color of these peppers is a consequence of their particular composition of chlorophyll (71%) and carotenoids (29%) [3]. Brown peppers (BPs) cannot develop a brilliant and homogeneous red color in postharvest due to the non-climacteric nature of pungent peppers [4]. Thus, BPs are not visually attractive to consumers, and therefore, they remain on local market displays until they become senescent and are discarded [5]. BPs are also discarded from several processing lines (e.g., pickling, smoking, cooking, etc.), most of them involving exposure to boiling water (8.5–17.5 min) and hot surfaces (150–210 °C for 10–20 min) [6,7].

Pectin is a plant polysaccharide highly demanded in the global market due to its importance as a functional ingredient (e.g., emulsifying, gelling, thickening, stabilizing, fat substituent, carrying, and delivering properties, etc.) in the food, biological, medical, and cosmetic industries [8]. Commercial pectin is obtained from citrus, apple, and sugar beet byproducts [9,10]. However, the availability of these common pectin sources is not expected to match the increasing demand for pectin, and new pectin sources are therefore needed [8,11]. Recently, Yue et al. [12] stated the urgency of exploring underutilized fruits as pectin sources to cope with the 8.6% annual growth of the global pectin market. BPs are in this category of fruits and have advantages over traditional pectin sources, which result from treating raw materials with different heating regimes or polysaccharide-degrading enzymes that alter the structure and, consequently, the functional properties of pectin [13,14]. These treatments cause alterations in pectin yield and main properties, especially in the molecular weight and degree of esterification [12,15,16]. This affects pectin functionality [8,9,10].

Research on properties of pepper pectin has been limited almost exclusively to pectin from green and red peppers [7,15,17,18]. Additionally, pectin from hot peppers has received less attention than that of sweet peppers, although interest in hot pepper pectin has increased in recent years [15,17,18,19,20,21,22]. Several pectin types have been extracted from green and red peppers and their byproducts and subsequently evaluated for chemical, physical, and functional properties [7,15,17,18,19,20,21,22]. It has been demonstrated that pectin is an important component of these peppers (~9.5–20.7% fresh pepper weight) and that its physicochemical and functional properties are comparable in many cases to those reported for pectin from common pectin sources [15,19,23]. However, the use of green and red peppers as a source of commercial pectin could not be justified due to the ethical concerns commonly involved in processes using foods to obtain products with technological applications [24]. Jalapeno BPs could be a source of commercial pectin as they are discarded or used for purposes other than consumption (i.e., seed production), and have a low value in the market [5]. Unfortunately, little is known about pectin from peppers at intermediate ripening stages, as it has been peripherally, dispersedly, and incompletely analyzed in studies related to pepper physiology [20,25,26]. To date, total and few pectin types (cold/hot water-, CDTA-, and EDTA-soluble pectins) have been quantified in a small number raw hot-pepper types at intermediated ripening stages and only some of them evaluated for molecular weight distribution, neutral sugar composition, and degree of esterification, although the later has only been evaluated in total pectin [25,26]. Other pectin types and characteristics have not been evaluated in these peppers.

Pectin from peppers at intermediate ripening stages must also be studied because the ripening process exerts a dramatic impact on several pectin properties (solubility, esterification degree, molecular weight, monosaccharide composition, etc.) [4,16,20,22]. The magnitude and velocity of these ripening-related changes in pectin strongly depend on the pepper type, as pectin structure and activities of pectin-degrading enzymes (polygalacturonase, α-L-arabinofuranosidase, etc.) significantly differ among them [27]. Recently, Zhao et al. [28] demonstrated that the physicochemical and functional properties of pectin vary significantly with pepper type, including aggregation capacity, hydrophobicity, molecular weight, degree of esterification, interfacial tension reduction ability, crosslinking capacity, and gelling and emulsifying properties. Currently, there is no research on the properties of pectin from Jalapeno BPs. The impact of heat processing of peppers at intermediate ripening stages on the physicochemical properties of their pectin also remains unexplored. However, studies on green and red peppers have demonstrated that heat treatment of peppers (e.g., pasteurization, blanching, boiling, grilling, etc.) causes significant alterations in pectin, including changes in pectin type composition, constituent sugars, linearity, degree of esterification, molecular weight, and several functional properties [7,15,22].

There is some evidence to hypothesize that Jalapeno BPs could be rich in pectin with physicochemical properties suitable for use as a functional ingredient. Firstly, some peppers at intermediate ripening stages are firmer, experience less firmness loss after heat processing (e.g., boiling and microwave processing, etc.), and have lower or similar activity of cell-wall degrading enzymes than green or red fruits [20,21,26,29]. This suggests good pectin integrity in these peppers, as firmness loss in peppers is closely related to pectin degradation [17]. Secondly, cell-wall materials or pectin from some pepper types at intermediate ripening stages have a similar content of uronic acids, neutral sugars, and molecular weight distribution to those of green and red fruits [20,25,26]. In some of these cases, the degree of methyl esterification of total pectin from peppers at intermediate ripening stages was either higher or similar to that of pectin from peppers at other ripening stages [20,25]. All of the above suggest potential advantages of BP as a pectin source over peppers at other ripening stages. Of course, there are studies suggesting the opposite, and therefore, the properties of pectin from each pepper type should be characterized at specific ripening stages. There is currently no study reporting all the basic physicochemical properties of three different pectin types from fruits of a specific pepper cultivar at intermediate ripening stage. Our study provides that information and additionally examines the impact of heat-processing of peppers on the properties of these pectins. Thus, the objective of the present study was to evaluate the basic properties of three pectin types from Jalapeno BP subjected to different heat treatments.

## 2. Materials and Methods

### 2.1. Plant Material

Jalapeno BPs (*Capsicum annuum* L.) were obtained from a commercial orchard in Chihuahua, Mexico. This pepper type has been essential in the Mexican diet for centuries, and it is currently the most produced, consumed, domestically marketed, and exported in the country [1,2,30]. BPs were harvested by pickers along with green peppers and recovered from field sacks. The fruit was distributed in nine groups, which were treated in triplicate with boiling water for 2.5 min (Blanching, B), boiling water for 12 min (mild heat, MHT), or placed on a hot plate at 210 °C for 12 min (intense heat, IHT). Blanched peppers were considered the raw control fruit. These processing conditions were selected because they can induce different degradation mechanisms of pectin (moist vs. dry heat) and represent the most common heat-processing styles for peppers in the country [31,32]. The temperatures and times for the specific treatment of BPs were determined by preliminary studies following the criteria established by Ornelas-Paz et al. [31]. These processing conditions are similar to those applied to peppers under domestic and industrial conditions [6]. BPs from each group were evaluated for weight, length, major diameter, dry matter, firmness, color, and pectin content and properties. Samples of all pepper groups were immediately subjected to pectin extraction, and the obtained pectin was immediately evaluated for constituent sugars, molecular weight distribution, degree of methyl esterification, protein content, color, and viscosity.

### 2.2. Extraction of Pectin

The peduncle of peppers was removed, and then, samples of whole peppers (500 g) were homogenized to a puree in a kitchen blender. Puree samples (500 g) were firstly depigmented with 96% ethanol (2 L) for 18 h at 4 °C and then six times with acetone (1.5 L) for 2 h at 25 °C. The solvent was discarded at each depigmentation step by filtration through filter paper. The retained solids (alcohol insoluble residues, AIR) were lyophilized, weighed, ground using a mortar and pestle, and stored under N_2_ at 4 °C until pectin extraction. Water, chelating agent, and alkali-soluble pectins (WSP, CSP, and NSP) were sequentially extracted from AIR samples (5 g), according to De Roeck et al. [33], with slight modifications. WSP was extracted from AIR using distilled hot water (500 mL, 96 °C, 5 min). CSP was extracted from the AIR remnant using 0.05 mol/L *trans*-1,2-diaminocyclohexane-N,N,N′,N′-tetraacetic acid monohydrate (CDTA) in 0.1 mol/L potassium acetate (500 mL, pH 6.5, 28 °C, 6 h). The pH of the solution used for CSP extraction was adjusted to 6.5 using 1N HCl or 1N NaOH, as needed. Finally, NSP was extracted from the AIR remnant with 0.05 mol/L sodium carbonate containing 0.02 mol/L NaBH_4_ (500 mL,16 h at 4 °C and then 6 h at 28 °C). The rate of AIR to extracting solution was always 1:100 (*w*/*v*; 5 g AIR in 500 mL of each extraction solution), which was within the range used (1:20–180, *w*/*v*) by others for pectin extraction [19,23,34]. Pepper puree depigmentation and pectin fraction extraction were always performed under agitation conditions using a VMS-C7 S1 stirrer (VWR Scientific, USA) at a power level of three. The pectin-rich extracts and solids (other fibers, OF) at each extraction step were separated by filtration, and their pH was neutralized with 1N HCl or 1N NaOH. The filtrates were mixed with 96% ethanol (ratio of 1:3, *v*/*v*; 0.5 L extract: 1.5 L 96% ethanol) and kept overnight at room temperature until pectin precipitation. The pectin was recovered by centrifugation (12,000× *g*, 4 °C, 5 min) and filtration. Filtration was always performed using Whatman paper No. 541 that had been dried at 70 °C until constant weight in order to consider the paper’s weight in AIR, pectin, and OF yields. Pectin and OF were washed with ethanol (3 L), lyophilized, weighed, dried at room temperature, ground with a mortar and pestle, and stored under N_2_. The yield of pectin and OF was reported as %. Pectin was characterized for its physicochemical properties. OF was evaluated for color, but it was not subjected to further analysis.

### 2.3. Analysis of Constituent Sugars

Sugars were released from pectin according to Garna et al. [34]. A pectin sample (100 mg) was mixed with 5 mL of 0.2 mol/L trifluoroacetic acid and heated at 80 °C for 72 h. Then, the sample was cooled, and its pH was adjusted to 5 with 14 mol/L NH_4_OH. Then, 100 µL of the enzymatic Macerex PM Complex (Enmex S.A. de C.V.; Tlalnepantla, Estado de Mexico, Mexico) was added, and the reaction was maintained at 50 °C for 24 h. The sample was heated at 96 °C for 3 min to stop the enzyme activity, cooled, filtered using a membrane of 0.22 µm pore size (Millipore Corp., Bedford, MA, USA), and injected (20 µL) into a Varian HPLC system. The extract was analyzed by HPLC using two different ion exchange columns and a refractive index detector (Star model 9040) (Varian, Inc., Palo Alto, CA, USA). Rhamnose (Rha), arabinose (Ara), and fucose (Fuc) were separated in a MetaCarb H^+^ Plus (7.8 mm × 30 cm, 5 µm) (Varian Inc.; Walnut Creek, CA, USA) ion exchange column at 58 °C using 0.0085 N H_2_SO_4_ as mobile phase (0.4 mL/min). Galactose (Gal), mannose (Man), and xylose (Xyl) were separated using a Supelcogel Pb ion exchange column (7.8 mm × 30 cm, 5 µm) (Sigma-Aldrich Corp.; St. Louis, MO, USA) at 70 °C using water (18.2 MΩ) as mobile phase at a flow rate of 0.5 mL/min. The quantification and identification of sugars were performed using standard compounds. Several molar mass ratios for constituent saccharides were determined according to previous studies [23,35] in order to obtain additional structural information about the tested pectins.

### 2.4. Determination of the Degree of Methyl Esterification of Pectin

The esterification degree of pectin was evaluated according to Ramos-Aguilar et al. [7]. A pectin sample (5 mg) was mixed with H_2_SO_4_ (2 mL) and water (1 mL) and allowed to react for 40 min at ambient temperature. The volume of the reaction was raised to 10 mL using water. Then, two aliquots (400 µL) of the mixture (hydrolyzed pectin) were separately mixed with 4 mol/L potassium sulfamate (pH = 1.6, 40 µL) and 75 mmol/L sodium tetraborate in concentrated H_2_SO_4_ (2.4 mL). The mixture was maintained at 96 °C for 20 min and then cooled. Then, 80 µL of a solution of *m*-hydroxydiphenyl (0.15% 3-phenylphenol in 0.5% NaOH) or 0.5% NaOH (control reaction) was added, and the content of galacturonic acid (GalA) was determined at 525 nm using a calibration curve constructed with GalA.

The methanol content in pectin was determined by HPLC after alkaline hydrolysis. Aqueous solutions of pectin (1.67%, *w*/*v*) were prepared. An aliquot of these solutions (600 µL) was mixed with 7 mol/L NaOH (100 µL) and isopropanol (700 µL), vortexed, and incubated at 25 °C for 2 h. Then, the supernatant of the mixture (800 µL) was recovered by centrifugation (12,000× *g* for 10 min) and mixed with concentrated H_2_SO_4_ (10 µL). The mixture was filtered and manually injected (20 µL) into the Varian HPLC system described above. The methanol was separated in a TSKgel SCX H^+^ (7.8 mm × 30 cm, 5 µm) cation exchange column (Tosoh Bioscience LLC; Tokyo, Japan) at 40 °C using water (18.2 MΩ) as mobile phase at a flow rate of 1 mL/min. The methanol was quantified using a calibration curve constructed with absolute methanol.

The degree of methyl esterification (DM) of pectin was determined as the ratio of mmol/L of methanol to mmol/L of GalA × 100.

### 2.5. Distribution of Molecular Weights

Aqueous solutions of pectin (0.50%, *w*/*v*) were filtered and injected (20 µL) into the Varian HPLC system described above. The pectin fractions were separated by high-performance size-exclusion chromatography in the TSKgel columns (TOSOH Bioscience; Minato-ku, Tokyo, Japan) GMPW_XL_ (7.8 mm × 30 cm, 13 µm), G5000PW_XL_ (7.8 mm × 30 cm, 10 µm), and G4000PW_XL_ (7.8 mm × 30 cm, 10 µm) connected in series at 40 °C [36]. The mobile phase was phosphate buffer (0.2 mol/L, pH 6.9) at a flow rate of 0.4 mL/min. The molecular weights were determined relative to dextrans, as performed by others [37,38].

### 2.6. Viscosity of Pectin Solutions

The pectin was dissolved in water under constant stirring at a final concentration of 2% (*w*/*v*). The viscosity of these solutions was determined at 25 °C using an AR 1500ex rheometer (TA Instruments; New Castle, DE, USA) equipped with stainless steel parallel plate geometry (60 mm diameter), as described by Ramos-Aguilar et al. [7]. The shear rate was from 0.01 s^−1^ to 500 s^−1^ (up curve) and from 500 s^−1^ to 0.01 s^−1^ (down curve). A gap size of 500 µm was set. The shear rate against shear stress data was fit using the power law model ($\tau =K{\dot{\gamma }}^{n}$) and analyzed for flow behavior index, *n*, and consistency index, *k*.

### 2.7. Other Measurements

Three subgroups of BPs (10 fruits each) from each experimental group were individually weighed and evaluated for length and major diameter using a vernier caliper. The firmness was determined in the pericarp of 10 fruits per group using a TA-XT2i texture analyzer (Stable Micro Systems Ltd., Godalming, UK) equipped with a 6 mm Ø stainless steel striker pin, which punctured half of the pericarp thickness at a rate of 10 mm/s. The maximum force (in N) needed to puncture the fruit was recorded. The dry matter content was gravimetrically determined in halves of 10 fruits, considering their weight before and after drying at 110 °C. The color (L*, a*, and b*) was determined directly on the pepper puree used for pectin extraction as well as on pectin and OF using a CR-300 model Minolta colorimeter (Minolta Co., Ltd., Osaka, Japan). Pectin was dissolved in water at a concentration of 0.5% (*w*/*v*) and evaluated in triplicate for protein content using the Bradford assay. The methodologies for these measurements have been described previously [3,7,39].

### 2.8. Statistical Analysis

The experiment was repeated three times under a completely randomized design. The data were analyzed by a one-way ANOVA followed by the Tukey–Kramer post hoc test using a value of 0.05 as the significance limit. All measurements were performed nine times, unless stated otherwise. Correlation analyses were also performed for the main chemical properties of pectin, considering the individual effects of pectin type and heat processing intensity. The data analysis was performed using JMP 11.0.0 statistical software (SAS Institute Inc.; Cary, NC, USA).

## 3. Results and Discussion

### 3.1. Pectin Yield

MHT caused only minimal changes in BP attributes compared to blanching. However, IHT caused significant changes in these attributes (Table 1). IHT caused a firmness loss of 82.6%, suggesting an important impact of heating on the integrity of cell wall materials [3].

Blanched chilies contained less AIR than samples treated with MHT and IHT (Table 2). Similar impacts of heat treatment of peppers and other pectin sources (coffee pulp, banana peels, etc.) on AIR yield have been reported previously [7,40,41]. This variation in AIR yield was driven by the heat treatment rather than by an incomplete inhibition of cell-wall degrading enzymes by blanching, as the total pectin content in AIR of blanched and MHT-treated peppers was the same (29.9% and 30.2%). Blanching caused greater variability in AIR yield compared to the other heating treatments, probably due to slight variations in the ripening stage of BPs, given the fast ripening-related modification of cell-wall materials reported for peppers [20,26], which resulted in measurable variations in AIR yield under low heating conditions. Higher intensity of heating could mask these variations by increasing AIR extractability.

On the other hand, the total pectin content in the tested peppers (11–14.6% of fresh pepper weight) was similar to that (~9.5–20.7% of fresh pepper weight) already reported for untreated green and red peppers [7,15,19]. However, these pectin contents are significantly higher than those reported for pepper byproducts [23]. This demonstrates that peppers are more suitable as a pectin source than pepper byproducts. The content of the three pectin types was almost similar in blanched BPs (Table 2). WSP was slightly more abundant than the other pectin types in blanched BPs, suggesting that the ripening-related pectin solubilization had already taken place [20]. MHT and IHT favored the yield of WSP with the concomitant decrease in CSP and NSP, suggesting the heat-mediated interconversion of pectin types. Heat treatment of peaches (100 °C) also caused a significant solubilization (~50%) of CSP [42]. These changes in the proportion of pectin types have been observed for other pectin sources and suggest heating-mediated pectin solubilization [41]. Increases in WSP and decreases in NSP have also been observed in green Jalapeno peppers, carrots, broccoli, and other plant foods after heat processing, mainly being attributed to the conversion of insoluble pectin and protopectin into soluble pectin by the thermo-solubilization and/or β-eliminative depolymerization of these materials [43,44,45]. The proportion of pectin types in plant tissues depends on many factors, including the source, pretreatment of the source, and extraction conditions (temperature, time, pH, extracting solvent type, etc.) [19,23]. The content of OF decreased with heat treatments, as reported previously for green and red peppers [7].

### 3.2. Constituent Sugars of Pectin

The content of GalA was higher in WSP than in the other pectin types, independently of the treatment (Table 3). WSP from untreated citrus pulp also contained more GalA than CSP and NSP [39]. This could be attributed to the solvents used for pectin extraction, which differently broke hydrogen bonds, ester linkages, and side chains (e.g., arabinans, galactose chains, etc.) from the main pectin chain, causing relative changes in GalA [19,46]. This inference is supported by the positive correlation (*p* ≤ 0.05) observed for the changes in GalA and Rha (r = 0.75–0.77) in WSP and CSP, independently of heat treatment intensity, as Rha favors pectin branching [47]. However, Ramos-Aguilar et al. [7] observed that WSP from green and red Jalapeno peppers contained less GalA than CSP and NSP. This suggests that ripening significantly affects sugar composition, including GalA, in pepper pectin. MHT reduced 9–23.7% of the GalA content in tested pectins, while IHT increased it (8.3–31.2%). Pectin solubilization, degradation, and leaching into the water used for MHT could cause GalA reduction in pectin from these peppers. Einhorn-Stoll and Kunzek [48] observed that increasing the ambient humidity from 65% to 85% at 60 °C was sufficient to decrease the galacturonan content in some pectins. Under water excess conditions, uronic acids tend to leach into the water, and pectin degrades by a β-elimination mechanism as the water temperature increases, as demonstrated for lentil pectin exposed to water at 25–100 °C [49]. On the other hand, IHT could favor the breaking of side chains (e.g., galactans, arabinans, etc.), causing a relative increase in GalA [19]. IHT could also favor the aggregation of pectin fragments, causing increases in GalA, as demonstrated for citrus pectin exposed to dry heat [50]. The treatment of sugar beet flakes with hot dry air (60 °C) also caused increases in the content of GalA in the extracted pectin compared to pectin from lyophilized flakes [51]. Ramos-Aguilar et al. [7] demonstrated that heating green pungent peppers decreased the GalA, while the opposite was observed for red peppers. Obodo-Ovie et al. [19] observed a decrease in GalA in green bell pepper pectin as the temperature of the aqueous solution used for pectin extraction increased from 60 °C to 80 °C. Mao et al. [41] also observed that the content of GalA in the pectin of banana peels at different stages of ripening decreased when the temperature for pectin extraction increased from 110 °C to 130 °C.

The contents of Man, Gal, and Ara were significantly higher than those of Xyl, Rha, and Fuc. Man, Gal, and Ara are also abundant in pectins from other plant sources, including citrus and apples, with pectin rich in arabinan chains showing an important gelling property in the presence of acids and Ca^2+^ [13,39,52]. The content of some of these sugars was differently affected by the applied heating regimes. Interestingly, the heat-mediated changes in Rha showed a negative correlation (*p* ≤ 0.05) with those of GalA (r = −0.89–−0.95), independent of pectin type, suggesting that heat treatment influenced the smooth-to-hairy regions ratio of pectin [53]. MHT and IHT increased Xyl in WSP and NSP, but Xyl decreased in CSP. The presence of Xyl demonstrates the co-extraction of pectin and hemicellulose. Zhang et al. [52] also observed increases and decreases in Xyl in apple pectin depending on the temperature (100–180 °C) and time (0–15 min) used for pectin extraction. Overall, heating decreased Gal and Man in all pectins. Obodo-Ovie et al. [19] also observed that the pectin from non-pungent green peppers extracted at pH 3 showed less Gal when the extraction process was performed at 80 °C instead of 60 °C. This heat-mediated decrease in Gal and Man might change pectin functionality, as chains of these and other sugars determine the hardness of pectin gels and other functions [54]. Decreases in neutral sugars have been related to the degradation process in side chains of pectin due to the harsh conditions used for the extraction or treatment of raw pectin sources [55]. Heat treatments either did not affect the content of the other sugars in pectin or caused minor changes, as observed previously for pectins from pungent and non-pungent green and red peppers [7,19].

The tested pectins were mostly composed of HG (86.5–96.7%) (Table 4). Similar HG or GalA contents (86.3–93.6%) have been reported for other pectins [56,57]. The high HG content was also confirmed by the high values observed for the molar mass ratio GalA/Rha. Obodo-Ovie et al. [19] and Xu et al. [23] also concluded that pectin from red hot peppers and green Bell peppers was mostly composed of HG. This suggests that our pectins were highly linear and that the most abundant glycosidic bond was α–(1→4). Overall, the homogalacturonic purity of the three pectins increased with the intensity of heat treatment, especially that of WSP and CSP (Table 4). This heat-mediated increase in homogalacturonic purity was mostly determined by the loss of Rha, Gal, and Ara, as revealed by the ratios GalA/(Rha + Gal + Ara) and GalA/Rha. Treatment of peppers with boiling water, independent of treatment time, favored the swelling and solubilization of side chains of arabinans and galactans from RG-I in WSP, according to (Gal + Ara)/Rha values, while IHT caused the degradation of these chains in the three pectin types. The ratios GalA/(Rha + Gal + Ara) and (Gal + Ara)/Rha suggest the presence of the glycosidic bonds α–(1→2), α–(1→5), and β–(1→4), which are characteristic of linkages GalA–Rha, Rha-Gal, and Rha-Ara in RG-I. These additional linkages were probably present at low abundance due to the high HG content in the pectins. On the other hand, the ratios Gal/(Rha + GalA) and Ara/(Rha + GalA) revealed that the length of galactan chains in RG-I was higher than that of arabinans in the three pectin types and that galactan chains were more susceptible to thermal degradation than arabinans. Overall, MHT favored the solubility of these side chains while IHT degraded them.

### 3.3. Degree of Methyl Esterification of Pectin

**Figure 1:**
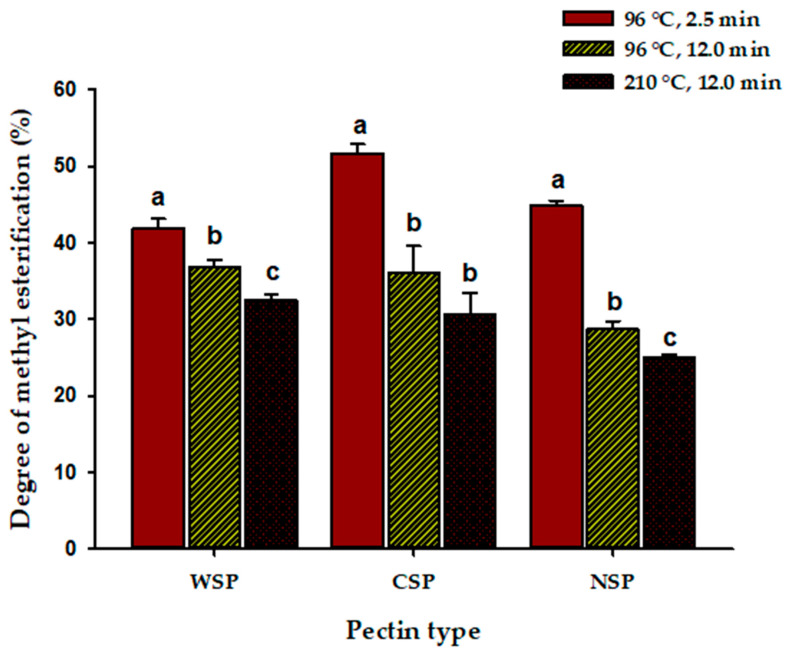
Degree of methyl esterification of water-, chelating-, and alkali-soluble pectin (WSP, CSP, and NSP) from brown Jalapeno peppers (BPs) after blanching (water at 96 °C for 2.5 min), mild heat treatment (water at 96 °C for 12 min, MHT), and intense heat treatment (hot plate at 210 °C for 12 min, IHT). Data represent the mean of nine replicates ± the standard error (slim bars). Bars with different letters for each pectin type were statistically different (*p* < 0.05).

The DM of pectins from blanched peppers ranged between 41.9% and 51.6% (Figure 1). These DM values were within the range reported generally for pectin from other chilies (DM = 40–67%) and other pectin sources, such as citrus (DM = 37.2–65.8%) and apples (DM = 22.8–67.4%) [13,23,39,58,59]. Overall, the extracted pectins are classified as low-methoxyl pectin, as only CSP from blanched peppers showed a DM slightly above 50%. However, the difference in DM for the three pectin types from blanched peppers was small (Figure 1). Recently, Bao et al. [15] also found that the pectin from Xiaomila chili peppers was, in general, of low DM and observed that CSP and WSP had higher DM than NSP, with this being attributed to the de-esterification of NSP during the extraction process. CSP from untreated citrus pulp also had higher DM than WSP and NSP [39]. CSP from bilberry also showed a higher DM (77%) than other pectin types [60]. Yoshioka et al. [61] demonstrated that polyuronides of high DM were commonly found in CSP. CSP from blanched BPs could be used, for example, to form gels in the presence of sugars and acids, according to its DM [14]. On the other hand, WSP and NSP (low DM) from blanched BPs could be used as a functional ingredient in restructured fish products due to their texturizing properties without significantly affecting the food color [62].

Pectins of low DM have good color-stabilizing properties in anthocyanin-pigmented solutions [63]. Pectin of low DM can be used as a gelling and thickening agent, and stabilizer against coalescence, creaming, and sedimentation in low-sugar or sugar-free foods (confectionery products, low-sugar jam and jellies, etc.). It can also be used as a fat replacer in dairy products due to the strong interaction between the anionic groups of pectin with the positively charged casein [64,65]. Some studies have demonstrated that pectins of low DM influence the texture and firmness of fruits because free carboxyl groups can be crosslinked with divalent ions such as Ca^2+^, forming a fortifying network [14]. Thus, the extracted pectin could be used to modify the texture of processed foods. In our study, the DM of CSP and NSP negatively correlated (*p* ≤ 0.05) with GalA (r = −0.92–−0.95), independently of heat treatment intensity, suggesting that the chelating and alkaline extracting solutions favored a greater number of free carboxyl groups in the galacturonic acid units, as reported for other pectins [53].

On the other hand, the DM of all pectin types from blanched peppers decreased sequentially with MHT and IHT. The highest heat-mediated de-esterification was observed for CSP (Figure 1). Sila et al. [66] also observed that the treatment of carrots with hot water (90 °C, 4 min) caused the highest demethylesterification in CSP, as compared with other pectin types. Xian et al. [42] observed that treating peaches with hot water at several temperatures caused significant decreases in the DM of WSP, CSP, and NSP. Interestingly, the impact of the heat treatment on DM of pectin not only depends on the pectin type, extraction conditions, and pectin source but also on the ripening stage of the pectin source, as demonstrated in banana peels and many other fruits and vegetables [41]. Pectin of high DM, as our CSP, is more susceptible to non-enzymatic degradation by β-elimination than pectin of low DM, explaining the effect of heating on this response variable [66]. The DM of pectin highly influences its function in plant materials and technological formulations [14,62,63]. Interestingly, blanching and MHT caused changes in DM that positively correlated (*p* = <0.05) with changes in Man (r = 0.79–0.84), independently of pectin type, suggesting the release of Man side chains from the main pectin chain as a consequence of these heat treatments and their subsequent leaching into the water used for treatment [67].

### 3.4. Molecular Weight of Pectin

Pectins from blanched pods contained two or three main fractions of molecular weight (MW), peaking between 1997 and 6 kDa (Table 5). These values of MW are in the range reported for pectin from green and red pungent peppers and their byproducts (3720–2.6 kDa), although pepper pectin has scarcely been fractionated according to its solubility and MW [7,15,23]. Similar MWs have also been reported for pectin from other sources (4092–2 kDa) [13,38,39]. Considering only the first fraction of pectin, which was the most abundant and largest, it can be concluded that NSP showed a higher MW than CSP and WSP. Bao et al. [15] also observed that NSP from Xiaomila pungent peppers had higher MW than WSP and CSP. Similar results were observed for carrot pectin [44], probably due to this pectin type is usually more branched and contains Ara and Gal chains [68], as observed in this study for NSP, which had the highest Gal content. The peak MW of the first fraction in all of the pectins depended on the content of several constituent sugars, especially Man (r = 0.84–0.96) and Gal (r = 0.41–0.92), independently of the intensity of the heat treatment, as observed in pectin from other sources [53]. This suggests that pectin from BPs contains side chains of Man and Gal. NSP from blanched peppers could form gels and films of high mechanical strength and elasticity and better water vapor barrier properties, given their high MW and branching [69,70].

MHT and IHT favored the formation of other pectin fractions of lower MW (Table 5), demonstrating that heating favored pectin depolymerization, as reported by others [31,64]. MHT reduced the MW of fraction I of WSP (13.5%), CSP (17.2%), and NSP (18.2%), while IHT reduced, even more, the MW of this fraction (22.6%, 86.2%, and 79.5% for WSP, CSP, and NSP, respectively). The peak MW of this fraction in pectin from blanched and MHT peppers showed a positive correlation (*p* ≤ 0.05) with changes in Rha (r = 0.89–0.92), indicating the influence of heat-mediated loss of side chains [58]. On the other hand, the decreases in the MW of this fraction in pectins from MHT and IHT peppers were also related to Man (r = 0.73–0.79) and, in the case of pectins from IHT peppers to Gal content (R = 0.87), independently of pectin type. These results confirm that BP pectin contains side chains, mostly of Man and Gal. Ramos-Aguilar et al. [7] also observed a significant and gradual decrease (8–98%) in the MW of the most abundant and largest fraction of WSP, CSP, and NSP as the intensity of the heat treatment applied to green and red peppers increased from mild to intense. Obodo-Ovie et al. [19] recently observed that the MW of pectin from green bell peppers (4096–812 kDa) strongly depended on the extraction conditions, including temperature, explaining the difference in the MW of fractions observed in our study for each pectin type as a function of the heat treatment. Raka et al. [71] observed that the heat treatment of sugar beet pectin (80 °C for 24 h) caused a dramatic decrease (by one order of magnitude) of the MW of pectin. In all cases, these reductions were attributed to heat-mediated depolymerization and overall pectin degradation [48,71]. In our study, CSP and NSP were particularly susceptible to heating-mediated depolymerization, probably due to monovalent ions of cell walls competed with divalent ions for interaction with free carboxyl groups in these pectin types, with this favoring depolymerization by heating [66]. However, the release of GalA due to heating (Table 3) and/or β-elimination degradation induced by high methoxy-ester content in CSP might also be involved. Bao et al. [15] observed the highest decreases in MW in NSP after heat treatment of pungent peppers.

MW of pectin has a substantial impact on its functionality, although pectin functionality depends on other structural characteristics like DM, protein content, GalA content, etc. The negative impact of heat processing on the MW of the tested pectins might compromise their gelling and emulsifying properties. It has been observed that the number of junctions and overall connectivity among pectin chains decrease in gels with the MW of pectin, leading to gels with reduced hardness, elasticity, and water-holding capacity [14,28,37,54,64]. On the other hand, low MW pectin is also used to show reduced emulsifying properties, as it adsorbs rapidly at the interface, forming thin interfacial layers with limited steric stabilization, favoring coalescence and flocculation [8,28,35,72]. However, pectins from heat-processed peppers might show some functionality in spite of their low MW if other of their properties are considered [10]. Overall, heat treatment decreased pectin DM and MW (Figure 1, Table 5) but increased GalA content (Table 3), which might improve some functional properties (emulsifying, thermal stability, antioxidant activity, bile acid binding capacity, etc.) [12]. Of course, these heating-mediated changes in pectin structure could also compromise other important functional properties (viscosity, gelling, flow properties, etc.) [12]. Further research is needed to characterize the functional properties of pectin from peppers at the intermediate ripening stage.

Our data are insufficient to determine the mechanisms involved in the depolymerization of BP pectin. However, we infer that pectins from blanched and MHT peppers were probably depolymerized by hydrolysis, as this mechanism is water-dependent and mainly causes the release of side chains, as observed in our study [32]. Water was abundant during blanching and MHT of peppers. On the other hand, depolymerization by β-elimination can occur in the absence of water; therefore, this depolymerization mechanism was probably the most important in IHT peppers [32]. However, our data suggest that several depolymerization mechanisms occurred in all treatments.

### 3.5. Protein Content of Pectin

The protein content in pectin from blanched BPs varied between 1.4% and 1.7% (Table 6). Slightly higher protein contents (1.9–2.7%) have been reported for WSP, CSP, and NSP from untreated citrus fruits [39]. In our study, NSP showed a higher protein content than the other pectins, as observed previously for NSP from pungent peppers (green and red) and citrus fruits [7,39]. NSP can be preferentially associated with proteins due to its low methoxylation [73].

On the other hand, tested heating regimes slightly increased the protein content in pectin (Table 6), probably due to the heating-mediated solubilization of pectin chains lacking protein. Ramos-Aguilar et al. [7] observed that heat treatment increased the protein content in WSP, CSP, and NSP from red peppers, while this effect was only observed clearly for NSP from green peppers. The emulsifying and stabilizing effects of pectins have been associated with their protein content [74]. Protein-rich pectin leads to improved interfacial activity and the formation of an inter-polymer network structure, which favors viscosity, thickening, and emulsifying properties of pectin [75]. The hydrophobicity of protein residues favors the emulsifying and surface-active properties of natural pectin [76]. However, a higher protein content in pectin might compromise its potential gelling properties because a higher protein content reduces hydrogen bonding between carboxyl groups during induced gelation [28].

### 3.6. Color of Pectin

The color of pectin can determine its technological uses. Pectin can be used, for example, as a functional ingredient in liquid formulations and films. Color and transparency of pectin-based films and liquid formulations are critical factors in marketing, consumer satisfaction, and product protection [77]. The concentration of pectin, processing temperature, and structural changes in pectin can lead to brownish or reddish shades and reduced transparency in pectin-based films and solutions [78]. However, the incorporation of bioactive compounds (e.g., anthocyanins, carotenoids, phenolic compounds, etc.) into the matrix of these films and solutions adds functional benefits, including the prevention of product degradation [79]. Pectin tends to degrade over time, which affects the color of products in which it is used. GalA degradation favors the development of a reddish color in fruit juices during storage [80]. Pectin contains phenolic compounds in its structure, such as procyanidins and ferulic acid, which bind preferentially to Ara and Gal chains [81]. The oxidation of these phenolic compounds leads to brown pigmentation. Pectin can also bind quinones resulting from the oxidation of phenolic compounds, significantly altering the color of pectin [81]. Carotenoids could also remain trapped in the pectin network during the extraction process, further affecting the color of pectin [55]. The involvement of carotenoids in pectin color is highly relevant in the case of peppers due to their high content of these pigments [82].

In general, the color of pectins from blanched peppers (L* = 81.6–88.2, a* = 0.89–0.98, b* = 15.7–17.5; Table 7) was similar to that reported for commercial citrus pectin (L* = 82.8–87.7, a* = 0.2–2.9, and b* = 13.0–18.4) [48]. The L* value of WSP was slightly reduced by MHT (6.3%), while IHT significantly reduced this color coordinate (14–27%) in all pectin types (Table 7). Thus, heating caused a darkening of pectin, affecting its potential technological uses. MHT caused decreases in a* in WSP and CSP, while IHT increased this color coordinate in all pectin types. IHT reduced the b* values in WSP and CSP, but this coordinate increased in NSP. Increases in b* have been considered the best indicator of pectin browning, which has been related to pectin demethylation [48]. Some studies have revealed that pectins with a high GalA content, low content of neutral sugars and minerals, and small quantities of xylogalacturonans improve the color-stabilizing properties of pectin [83].

### 3.7. Viscosity

**Figure 2:**
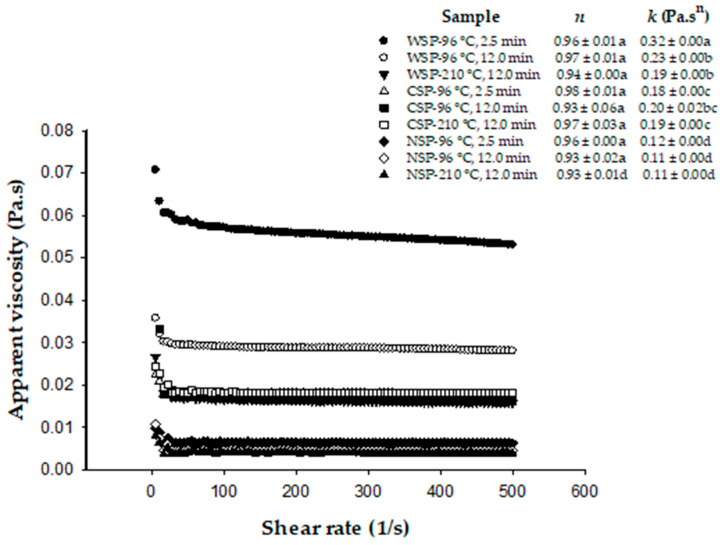
Representative flow curves and parameters of the power law model obtained from solutions of water-, chelating-, and alkali-soluble pectin (WSP, CSP, and NSP) from brown jalapeno peppers (BPs) after blanching (water at 96 °C for 2.5 min), mild heat treatment (water at 96 °C for 12 min, MHT), and intense heat treatment (hot plate at 210 °C for 12 min, IHT). *n*: flow behavior index. *k*: consistency index. Values in the same row with different letters are significantly different (*p* < 0.05).

The apparent viscosity of tested pectin solutions decreased as the shear rate increased (Figure 2), revealing a pseudoplastic nature of these fluids. The flow behavior was highly correlated (R^2^ = 0.92–0.98) with the power law model, with *n* values close to 1. All pectin solutions showed similar *n* values, suggesting that all pectins could also have the same ability for gel formation because it is expected a similar interaction force between the pectin molecules in the solution, favoring the formation of a similar quantity of “egg-box” junction zones [14]. The obtained *n* values were also expected partly due to the low concentration of pectin in the tested solutions.

The consistency index (*k*), which is an indicator of viscosity for power-law fluids [14], ranged from 0.12 Pa.s^n^ to 0.32 Pa.s^n^ for pectin from blanched BPs (Figure 2). These *k* values were similar to those reported (0.15–0.39 Pa.s^n^) for solutions of the same pectin types from apples and green and red pungent peppers [7,84] but slightly lower than those of the same pectin types from citrus fruits [39]. Our *k* values yielded apparent viscosities at shear rates below 1/s that were more than 100 times higher than that of water (0.001 Pa·s^n^). Our *k* values were also similar to those (0.1–0.26 Pa·s^n^) recently reported for apple pectin solutions (pH 5) containing a higher pectin concentration than that used in our study [81]. Thus, potential functional properties can be inferred for the tested pectins. WSP showed the highest *k* values as compared to CSP and NSP. Similar results were observed for these pectin types from okra [72]. In our study, the *k* of WSP solutions decreased (28–41%) with the intensity of heat treatment. The impact of heat treatment on *k* values was not observed clearly for CSP and NSP. Ramos-Aguilar et al. [7] also observed that the heat treatment of green and red pungent peppers caused higher decreases in *k* in solutions of WSP as the intensity of the heat treatment increased, while this effect was not observed clearly for solutions of CSP and NSP. *k* values did not relate well to MW and DM of pectins, as observed for pectins from other sources [14,16,72]. This is expected as the viscosity of pectin solutions depends on many factors, including the pectin source, molecular weight, aggregation degree, conformation of molecules, degree of esterification, pH, and temperature [14].

## 4. Conclusions

Our study provided new insights into the properties of pectin from peppers at intermediate ripening stages and how heat processing of peppers affects those properties. Overall, the rheological and physicochemical properties of brown Jalapeno peppers’ pectin are comparable to those reported for citrus, apple, and sugar beet pectins, which are the most important in the global pectin market. Heat treatment of these peppers increased the yield of water-soluble pectin but decreased the molecular weight, degree of methyl esterification, and contents of galactose and mannose in all tested pectins. Solutions of water-soluble pectin showed the highest viscosity. Interestingly, the heat treatment increased the relative content of protein in the tested pectins. Fresh and heat-treated brown Jalapeno peppers may be used as an alternative source of pectin with a low degree of methyl esterification. This research valorizes an overlooked agricultural product. Further research is needed to determine additional structural characteristics and the functional properties of the tested pectins under application conditions. Research is also needed to design practical and environmentally friendly strategies for extracting pectin from brown Jalapeno peppers without altering its chemical properties, as occurs with the widely used acid-based method. Strategies are also needed to scale up the pectin extraction process to an industrial level.

## Figures and Tables

**Table 1:** Attributes of brown Jalapeno peppers (BPs) after blanching (water at 96 °C for 2.5 min), mild heat treatment (water at 96 °C for 12 min, MHT), and intense heat treatment (hot plate at 210 °C for 12 min, IHT).

Attribute	Heat Treatment		
	Blanching	MHT	IHT
**Weight (g)**	25.3 ± 1.0 ^a^	25.7 ± 1.5 ^a^	17.7 ± 1.0 ^b^
**Length (cm)**	7.4 ± 0.2 ^a^	7.1 ± 0.2 ^a^	6.9 ± 0.2 ^a^
**Major diameter (cm)**	2.9 ± 0.0 ^a^	2.6 ± 0.1 ^b^	2.5 ± 0.1 ^b^
**Dry matter (%)**	12.5 ± 0.2 ^b^	12.0 ± 0.2 ^b^	16.4 ± 0.1 ^a^
**Firmness (N)**	39.1 ± 0.9 ^a^	10.9 ± 0.4 ^b^	6.8 ± 0.5 ^c^
Color *			
L*	34.6 ± 0.6 ^b^	39.3 ± 0.5 ^a^	24.0 ± 0.7 ^b^
a*	7.1 ± 0.3 ^b^	10.5 ± 0.3 ^a^	29.9 ± 0.3 ^a^
b*	30.5 ± 0.4 ^a^	8.0 ± 0.3 ^b^	22.7 ± 0.7 ^b^

Values represent the mean of 30 individual measurements± the standard error. Values in the same row for each attribute with different letters are significantly different (*p* < 0.05). * color of pureed peppers.

**Table 2:** The yield of alcohol-insoluble residues (AIR), water-soluble pectin, chelating-soluble pectin, and alkali-soluble pectin (WSP, CSP, and NSP), and other fibers (OF) from brown Jalapeno peppers (BPs) after blanching (water at 96 °C for 2.5 min), mild heat treatment (water at 96 °C for 12 min, MHT), and intense heat treatment (hot plate at 210 °C for 12 min, IHT).

Fiber Type	Heat Treatment		
	Blanching	MHT	IHT
**AIR (g/kg pepper)**	367 ± 22 ^b^	483 ± 9 ^a^	445 ± 10 ^a^
**Fiber (% AIR)**			
**WSP**	10.6 ± 1.4 ^b^	15.5 ± 0.2 ^a^	13.5 ± 0.9 ^ab^
**CSP**	10.0 ± 0.7 ^a^	6.3 ± 0.8 ^b^	6.7 ± 0.7 ^b^
**NSP**	9.3 ± 0.2 ^a^	8.4 ± 1.4 ^ab^	5.5 ± 0.2 ^b^
**OF**	65.4 ± 2.8 ^a^	56.6 ± 0.1 ^b^	57.5 ± 1.3 ^b^

Values represent the mean of nine measurements ± the standard error. Values in the same row with different letters are significantly different (*p* < 0.05).

**Table 3:** Content of constituent sugars in water-, chelating-, and alkali-soluble pectin (WSP, CSP, and NSP) from brown Jalapeno peppers (BPs) after blanching (water at 96 °C for 2.5 min), mild heat treatment (water at 96 °C for 12 min, MHT), and intense heat treatment (hot plate at 210 °C for 12 min, IHT).

Pectin Type	Heat Treatment/Compound Content (g/kg of Pectin)		
	Blanching	MHT	IHT
	***Galacturonic acid***		
**WSP**	796 ± 36 ^a^	724 ± 46 ^a^	862 ± 35 ^a^
**CSP**	569 ± 15 ^b^	434 ± 21 ^c^	695 ± 10 ^a^
**NSP**	462 ± 43 ^b^	367 ± 18 ^b^	606 ± 10 ^a^
	***Xylose***		
**WSP**	8.0 ± 0.3 ^c^	122 ± 2 ^b^	129 ± 1 ^a^
**CSP**	5.4 ± 0.1 ^a^	1.9 ± 0.1 ^b^	1.4 ± 0.1 ^c^
**NSP**	7.0 ± 0.1 ^c^	41.0 ± 0.1 ^a^	22.0 ± 0.4 ^b^
	***Galactose***		
**WSP**	50 ± 1 ^a^	26 ± 1 ^c^	42 ± 3 ^b^
**CSP**	31.0 ± 0.1 ^a^	16 ± 3 ^b^	14 ± 3 ^b^
**NSP**	95 ± 2 ^b^	105 ± 2 ^a^	45.0 ± 0.5 ^c^
	***Mannose***		
**WSP**	96 ± 3 ^a^	13 ± 2 ^b^	19 ± 2 ^b^
**CSP**	130 ± 2 ^a^	29 ± 3 ^b^	9 ± 1 ^c^
**NSP**	22 ± 1 ^a^	22.0 ± 0.1 ^a^	17 ± 2 ^a^
	***Arabinose***		
**WSP**	16.4 ± 0.2 ^a^	15.6 ± 0.4 ^ab^	15.0 ± 0.1 ^b^
**CSP**	13.0 ± 0.2 ^a^	15.2 ± 0.2 ^a^	15.3 ± 1.3 ^a^
**NSP**	18.0 ± 0.8 ^b^	24.1 ± 0.1 ^a^	26.9 ± 1.3 ^a^
	***Rhamnose***		
**WSP**	0.70 ± 0.02 ^b^	0.4 ± 0.1 ^b^	1.1 ± 0.1 ^a^
**CSP**	1.6 ± 0.1 ^a^	1.5 ± 0.1 ^a^	1.8 ± 0.1 ^a^
**NSP**	2.5 ± 0.1 ^a^	2.7 ± 0.1 ^a^	2.7 ± 0.3 ^a^
	***Fucose***		
**WSP**	0.30 ± 0.02 ^a^	0.34 ± 0.01 ^a^	0.30 ± 0.01 ^a^
**CSP**	0.39 ± 0.00 ^b^	0.42 ± 0.01 ^a^	0.38 ± 0.01 ^b^
**NSP**	0.49 ± 0.02 ^a^	0.52 ± 0.01 ^a^	0.37 ± 0.03 ^b^

Values represent the mean of nine individual measurements + the standard error. Values in the same row with different letters are significantly different (*p* < 0.05).

**Table 4:** Molar mass ratios for constituent saccharides in water-, chelating-, and alkali-soluble pectin (WSP, CSP, and NSP) from brown Jalapeno peppers (BPs) after blanching (water at 96 °C for 2.5 min), mild heat treatment (water at 96 °C for 12 min, MHT), and intense heat treatment (hot plate at 210 °C for 12 min, IHT).

Pectin Type	Heat Treatment/Molar Mass Ratio		
	Blanching	MHT	IHT
	**HG (%)**		
**WSP**	93.2 ± 0.1 ^c^	95.8 ± 0.2 ^a^	94.1 ± 0.3 ^b^
**CSP**	95.2 ± 0.0 ^b^	96.5 ± 0.3 ^a^	96.7 ± 0.2 ^a^
**NSP**	88.3 ± 0.2 ^b^	86.5 ± 0.2 ^c^	92.3 ± 0.1 ^a^
	**GalA/(Rha + Gal + Ara)**		
**WSP**	10.5 ± 0.5 ^b^	14.9 ± 0.4 ^a^	13.2 ± 1.0 ^ab^
**CSP**	11.0 ± 0.3 ^b^	11.2 ± 0.9 ^b^	19.0 ± 1.2 ^a^
**NSP**	3.6 ± 0.4 ^b^	2.5 ± 0.1 ^c^	7.0 ± 0.1 ^a^
	**(Gal + Ara)/Rha**		
**WSP**	93.2 ± 4.3 ^ab^	102.4 ± 14.8 ^a^	52.5 ± 7.8 ^b^
**CSP**	26.3 ± 0.2 ^a^	21.3 ± 2.9 ^ab^	16.1 ± 1.0 ^b^
**NSP**	42.9 ± 1.5 ^a^	45.8 ± 1.5 ^a^	26.2 ± 3.0 ^b^
	**GalA/(Gal + Ara + Rha + Xyl)**		
**WSP**	9.4 ± 0.6 ^a^	3.5 ± 0.2 ^b^	3.7 ± 0.2 ^b^
**CSP**	9.3 ± 0.2 ^b^	9.6 ± 0.3 ^b^	15.8 ± 1.5 ^a^
**NSP**	3.4 ± 0.4 ^b^	1.9 ± 0.1 ^b^	6.1 ± 0.8 ^a^
	**(Gal + Ara)/(Rha + GalA)**		
**WSP**	0.095 ± 0.005 ^a^	0.066 ± 0.002 ^b^	0.075 ± 0.006 ^b^
**CSP**	0.088 ± 0.002 ^a^	0.086 ± 0.007 ^a^	0.050 ± 0.003 ^b^
**NSP**	0.274 ± 0.024 ^b^	0.392 ± 0.018 ^a^	0.136 ± 0.003 ^c^
	**Gal/(Rha + GalA)**		
**WSP**	0.068 ± 0.004 ^a^	0.039 ± 0.001 ^b^	0.053 ± 0.005 ^ab^
**CSP**	0.058 ± 0.001 ^a^	0.040 ± 0.007 ^ab^	0.022 ± 0.004 ^b^
**NSP**	0.223 ± 0.022 ^b^	0.307 ± 0.015 ^a^	0.079 ± 0.001 ^c^
	**Ara/(Rha + GalA)**		
**WSP**	0.027 ± 0.001 ^ab^	0.028 ± 0.001 ^a^	0.023 ± 0.001 ^b^
**CSP**	0.030 ± 0.001 ^b^	0.045 ± 0.003 ^a^	0.028 ± 0.003 ^b^
**NSP**	0.051 ± 0.003 ^b^	0.085 ± 0.004 ^a^	0.057 ±0.003 ^b^
		**GalA/Rha**	
**WSP**	984.3 ± 52.8 ^ab^	1539.9 ± 219.9 ^a^	689.8 ± 60.7 ^b^
**CSP**	299.5 ± 7.6 ^ab^	244.5 ± 16.3 ^b^	321.6 ± 14.9 ^a^
**NSP**	159.4 ± 21.1 ^ab^	116.6 ± 9.0 ^b^	190.7 ± 18.5 ^a^

Values represent the mean of nine individual measurements + the standard error. Values in the same row with different letters are significantly different (*p* < 0.05).

**Table 5:** Peak molecular weight of main fractions in water-, chelating-, and alkali-soluble pectin (WSP, CSP, and NSP) from brown Jalapeno peppers (BPs) after blanching (water at 96 °C for 2.5 min), mild heat treatment (water at 96 °C for 12 min, MHT), and intense heat treatment (hot plate at 210 °C for 12 min, IHT).

Pectin Type	Heat Treatment	Pectin Fraction (Molecular Weight, kDa)			
		I	II	III	IV
**WSP**					
	**Blanching**	570 ± 10 ^a^	-	6.0 ± 0.1 ^a^	-
	**MHT**	493 ± 10 ^b^	-	6.0 ± 0.1 ^a^	-
	**IHT**	441 ± 6 ^c^	71 ± 2	5.0 ± 0.1 ^b^	-
**CSP**					
	**Blanching**	1813 ± 37 ^a^	-	-	8.0 ± 0.7 ^a^
	**MHT**	1501 ± 29 ^b^	472 ± 18 ^a^	134 ± 4 ^a^	7.0 ± 0.7 ^a^
	**IHT**	250 ± 21 ^c^	30 ± 1 ^b^	12.0 ± 0.2 ^b^	-
**NSP**					
	**Blanching**	1997 ± 13 ^a^	136 ± 2 ^a^	-	6.0 ± 0.1 ^a^
	**MHT**	1633 ± 18 ^b^	132 ± 2 ^a^	-	6.0 ± 0.1 ^a^
	**IHT**	409 ± 19 ^c^	30 ± 3 ^b^	8.0 ± 0.3 ^a^	-

Values represent the mean of nine individual measurements ± the standard error. Values in the same column for each pectin type and fraction (I–V) with different letters are significantly different (*p* < 0.05).

**Table 6:** Protein content in water-, chelating-, and alkali-soluble pectin (WSP, CSP, and NSP) from brown Jalapeno peppers (BPs) after blanching (water at 96 °C for 2.5 min), mild heat treatment (water at 96 °C for 12 min, MHT), and intense heat treatment (hot plate at 210 °C for 12 min, IHT).

Pectin Type	Heat Treatment/Protein Content (%)		
	Blanching	MHT	IHT
**WSP**	1.5 ± 0.1 ^a^	1.6 ± 0.1 ^a^	1.7 ± 0.7 ^a^
**CSP**	1.4 ± 0.1 ^b^	1.5 ± 0.1 ^ab^	1.6 ± 0.1 ^a^
**NSP**	1.7 ± 0.1 ^b^	2.0 ± 0.1 ^ab^	2.3 ± 0.1 ^a^

Values represent the mean of nine measurements ± the standard error. Values in the same row with different letters are significantly different (*p* < 0.05).

**Table 7:** Color of water-, chelating-, and alkali-soluble pectin (WSP, CSP, and NSP) and other fibers (OF) from brown Jalapeno peppers (BPs) after blanching (water at 96 °C for 2.5 min), mild heat treatment (water at 96 °C for 12 min, MHT), and intense heat treatment (hot plate at 210 °C for 12 min, IHT).

Pectin Type	Color Coordinate	Heat Treatment		
		Blanching	MHT	IHT
**WSP**				
	L*	81.6 ± 0.3 ^a^	76.5 ± 0.3 ^b^	59.6 ± 1.4 ^c^
	a*	0.98 ± 0.04 ^b^	−0.10 ± 0.20 ^c^	4.6 ± 0.2 ^a^
	b*	17.5 ± 0.1 ^a^	18.4 ± 0.2 ^a^	13.6 ± 0.5 ^b^
**CSP**				
	L*	88.2 ± 0.4 ^a^	85.2 ± 0.8 ^a^	75.5 ± 1.3 ^b^
	a*	0.89 ± 0.03 ^b^	−0.50 ± 0.04 ^c^	2.4 ± 0.2 ^a^
	b*	15.7 ± 0.1 ^a^	12.4 ± 0.4 ^b^	12.6 ± 0.3 ^b^
**NSP**				
	L*	86.1 ± 0.6 ^a^	84.4 ± 0.5 ^a^	63.8 ± 1.8 ^b^
	a*	−0.52 ± 0.04 ^b^	−0.51 ± 0.05 ^b^	4.3 ± 0.2 ^a^
	b*	13.2 ± 0.3 ^b^	13.7 ± 0.3 ^b^	15.1 ± 0.3 ^a^
**OF**				
	L*	88.2 ± 0.4 ^a^	86.1 ± 0.2 ^b^	72.5 ± 0.4 ^c^
	a*	0.08 ± 0.09 ^b^	−0.56 ± 0.04 ^c^	0.7 ± 0.03 ^a^
	b*	15.7 ± 0.4 ^a^	14.4 ± 0.1 ^b^	10.9 ± 0.2 ^c^

Values represent the mean of nine measurements ± the standard error. Values in the same row with different letters are significantly different (*p* < 0.05).

## Data Availability

The original contributions presented in this study are included in the article. Further inquiries can be directed to the corresponding author.
